# Influence of Sex on Suicidal Phenotypes in Affective Disorder Patients with Traumatic Childhood Experiences

**DOI:** 10.1371/journal.pone.0137763

**Published:** 2015-09-14

**Authors:** Alexandra Bernegger, Klemens Kienesberger, Laura Carlberg, Patrick Swoboda, Birgit Ludwig, Romina Koller, Nestor D. Kapusta, Martin Aigner, Helmuth Haslacher, Michaela Schmöger, Siegfried Kasper, Alexandra Schosser

**Affiliations:** 1 Department of Psychiatry and Psychotherapy, Medical University of Vienna, Vienna, Austria; 2 Department of Psychoanalysis and Psychotherapy, Medical University of Vienna, Vienna, Austria; 3 University Hospital Tulln, Department of Psychiatry, Tulln, Austria; 4 Department of Laboratory Medicine, Medical University of Vienna, Vienna, Austria; 5 Department of Neurology, Medical University of Vienna, Vienna, Austria; 6 Zentrum für Seelische Gesundheit LEOpoldau, BBRZ-Med, Vienna, Austria; Catholic University of Sacred Heart of Rome, ITALY

## Abstract

**Objectives:**

In the current study, we aimed to investigate the impact of childhood trauma on suicidal behaviour phenotypes in a group of patients with diagnosed affective disorder (unipolar or bipolar affective disorder).

**Patients and Methods:**

Patients with and without a history of childhood abuse, measured by Childhood Trauma Questionnaire (CTQ), were assessed to explore risks for suicidal behaviour (including suicide attempt, self-harm and non-suicidal self-injury). The tested sample consisted of 258 patients (111 males and 147 females, in-patients and out-patients at the Department of Psychiatry and Psychotherapy, Medical University of Vienna and University Hospital Tulln, Lower Austria). Psychiatric diagnoses were derived from the SCAN (Schedules for Clinical Assessment in Neuropsychiatry) interview. In addition, patients were administered the Lifetime Parasuicidal Count (LPC), Suicidal Behaviour Questionnaire (SBQ-R), and Viennese Suicide Risk Assessment Scale (VISURIAS) questionnaires.

**Results:**

In contrast to male suicide attempters, female suicide attempters showed both significantly higher total CTQ scores (p<0.001), and higher CTQ subscores (emotional, physical and sexual abuse, as well as emotional and physical neglect) in comparison to the non-suicidal control group. Besides, females with a history of self-harming behaviour (including suicidal intention) and Non-Suicidal-Self Injury (NSSI) had significantly higher CTQ total scores (p<0.001) than the control group.

**Conclusion:**

These findings suggest gender differences in suicidal behaviour after being exposed to childhood trauma.

## Introduction

Suicide is a still increasing major public health issue. Every year, more than 800,000 people all over the world commit suicide.[1] It is well known that 70–90% of completed suicides are preceded by mental disorders, mostly affective disorders [2, 3], and patients suffering from major depressive disorder (MDD) have an estimated 6–15% lifetime risk of suicide, [4] indicating the importance of adequate biological treatment of major depressive disorder. [5] Ten per cent of suicide attempters die due to suicide within 10 years. [6]

Risks for suicidal behaviour have been described by distal and proximal factors. Distal factors define the predisposing diathesis and determine the response of an individual to a proximal stressor. This group of distal factors includes variables as development, personality traits as well as biological and genetic variables. Proximal factors, which include life events, stress, psychiatric illness, alcohol or substance abuse, increase the individual risk on the basis of the distally determined threshold. [7, 8]

Furthermore, childhood trauma has been described as an important and independent risk factor for suicidal behaviour. [9–11] Clinical studies have pointed to an impact of childhood trauma on suicide attempts in different psychiatric disorders like schizophrenia, unipolar depression and bipolar disorder. [12–14] A Korean study showed that the prevalence of suicidal behaviour among students with continuous emotional abuse was 34% compared to 18% among those with no trauma in their history. [15] A recently published meta-analysis on childhood sexual abuse and suicidal behaviour described a direct association between childhood sexual abuse and later suicide attempts after controlling for genetic factors, family environment and other risk factors. [16]

Moreover, previous gene-environment (GxE) studies showed interactions between childhood trauma and defined genotypes of the 5-HTTLPR (serotonin-transporter-linked polymorphic region)-gene, as well as the CRHBP (Corticotropin-releasing factor-binding protein)—and FKBP5 (FK506 binding protein 5)-gene. [17, 18]

Suicidal behaviour is a complex phenotype. Self-harm includes behaviour directed to oneself with or without intent to die, whereas Non-Suicidal-Self-Injury (NSSI) is defined as intentionally damaging a part of the body without a conscious intent to die. [19] The differentiation of these phenotypes is of clinical importance, since patients treated in a hospital due to injuries resulting from self-harm have an up to 100-fold greater risk of completed suicide as compared to the general population. [20]

The aim of this study was to assess childhood abuse and its possible effect on subtypes of suicidal behaviour in consideration of gender. We wanted to test the hypothesis that different types of childhood trauma result in a distinct suicidal phenotype like suicide attempt, non-suicidal self-injury (NSSI) or self harm behaviour.

## Subjects and Methods

In total, 258 patients with either depressive disorder or bipolar affective disorder were examined at the General Hospital of Vienna (AKH, Medical University of Vienna) or the University Hospital Tulln (UK Tulln, Lower Austria). Diagnoses were defined by the research criteria of ICD-10 (World Health Organisation 1993) and/or DSM-IV-TR (American Psychiatric Association 2000), assessed by a structured interview using the Schedules for Clinical Assessment in Neuropsychiatry (SCAN) [21]. Inclusion criteria were age between 18–65 (or older if otherwise healthy), male or female, outpatients or inpatients. Exclusion criteria were: a personal psychiatric history of schizophrenia, mood incongruent psychotic symptoms, primary substance abuse, a primary organic disease, pregnancy and breast-feeding.

Before participation in the study, patients were informed in detail about the research goals of the study and also received written information with all relevant data. All patients signed a written consent form. The participants’ capacity to consent was assessed by the attending psychiatrist. Approval for the study was obtained from the Ethical Committee of the Medical University of Vienna (approval number EK 2013/2013) and the Ethical Committee of the federal state of Lower Austria (approval number GS4- EK-4/181/2012).

Raters assessed lifetime suicidal behaviour using three different questionnaires: suicide attempts and Non-Suicidal Self-Injury (NSSI) were assessed by the VI-SURIAS (Viennese Suicide Risk Assessment Scale) [22] and the German translation of the SBQ-R (Suicide Behaviour Questionnaire Revised). The SBQ-R is a brief self-report questionnaire containing 4 items, each assessing different dimensions of suicidality in order to clinically identify at-risk individuals and specific risk behaviours. [23] VI-SURIAS is an assessment scale containing 3 items (lifetime risk factors, previous events (last 6 month) and immediate risk factors [within the last week]) to clinically evaluate suicide risk and to define preemptive measures. [22]

Self-harm was assessed applying the LPC (Lifetime Parasuicidal Count), a questionnaire measuring the lifetime history of number of self-injurious behaviours grouped by method, intend to die and level of medical treatment.[24] Patients also completed a German version of the 28-item Childhood Trauma Questionnaire, for which the reliability had been proved. [25] This self-report questionnaire consists of 5 subscores (a 5-point Likert scale each, to rate for incidents from 1 = “never true” to 5 = “very often true”): emotional abuse, physical abuse, sexual abuse, emotional neglect and physical neglect. Each subscale consists of 5 questions, resulting in score ranges from 5 (no childhood maltreatment) to 25 (extreme history of maltreatment and abuse). Three questions assess the tendency to minimize or deny abuse and neglect. Cut-off scores determining abuse as yes/no have been set for each subscale: emotional abuse 13, physical abuse 10, sexual abuse 8, emotional neglect 15 and physical neglect 10. [26–28]

Psychiatric interviews were performed by a trained team of raters under the supervision of the PI (A. S.).

Statistical analyses were conducted using SPSS 22.0 (IBM, Armonk USA). Prior to the study, the appropriate sample size was estimated using G*Power 3.1. [29] Differences exceeding the internal reliability of the test (Cronbach α = 0.94, [25]) were considered as clinical relevant, which corresponds to an effect size of Cohen’s d = 0.5 and can be interpreted as a medium effect. Assuming a n_2_/n_1_-ratio of 4 and accepting an α-error of 0.05 and a β-error of 0.2 (power = 0.8), those effects would be detectable at a sample size ≥208. Continuous data were presented as mean and standard deviation, respectively confidence interval. Qualitative data were given as counts and percentages. In between group differences were computed using Fisher’s exact test and χ2 analysis for categorical variables. Differences between groups were assessed by means of One-way ANOVA testing for variables following a Gaussian distribution, or Mann-Whitney U Test for skewed data. A binary logistic regression model was established to account for potential confounders such as sex, martial status and employment. Gaussian distribution of the variables was tested by the Kolmogorov-Smirnov test. All test results were interpreted two tailed with a significance level established at p<0.05. Multiple testing corrections were performed for single Mann-Whitney U-tests by application of the false discovery rate. [30]

## Results

### 3.1 Characteristics of the sample

Descriptive statistics are presented in Tables 1 and 2. In total, 258 patients were interviewed, 3 patients had to be excluded from further analysis, because the interview could not be finished. Two hundred eleven patients (82.7%) were diagnosed with major depressive disorder (MDD) and 44 patients (17.3%) with bipolar disorder (BP). Seventy patients (27.5%) have attempted to commit suicide at least once in lifetime. Out of 91 (35.7%) patients presenting with a history of self-harm, 48 patients (18.8%) undertook Non-Suicidal Self-Injury (NSSI) actions and therefore had no intent to die. Seventy patients (27.5%) reported recurring transient suicidal thoughts and 64 patients (25.1%) had concrete suicidal thoughts.

**Table 1:** Social demographic and clinical characteristics of the patient sample.

	whole sample (n = 255)	male (n = 110)	female (n = 145)	χ2 and p-value
**MDD**	211 (82.7%)	90 (81.8%)	121 (83.4%)	χ2 = 0.1 p = 0.741
**BD**	44 (17.3%)	20 (18.2%)	24 (16.6%)	χ2 = 0.1 p = 0.741
**suicide attempt**	70 (27.5)%	25 (22.7%)	45 (31.0%)	χ2 = 2.2 p = 0.158
**self-harm**	91 (35.7%)	33 (30.0%)	58 (40.0%)	χ2 = 2.7 p = 0.114
**non suicidal self-injury (NSSI)**	48 (18.8%)	13 (11.8%)	35 (24.1%)	χ2 = 5.6 p = 0.022
**transient suicidal thoughts**	70 (27.5%)	38 (34.5%)	32 (22.1%)	χ2 = 4.88 p = 0.033
**concrete suicidal thoughts**	64 (25.1%)	26 (23.6%)	38 (26.2%)	χ2 = 0.2 p = 0.664

MDD (Major Depressive Disorder), BD (bipolar disorder), p-value in squared bracket = FDR corrected p-value.

**Table 2:** Childhood Trauma Scores of the total patient sample.

	whole sample (n = 255)	male (n = 110)	female (n = 145)	p	U
**CTQ total score M±SD**	52.4 ± 20.1	49.5 ± 18.4	55.8 ± 21.6	0.06	-0.19
**CTQ emotional abuse**	10.0 ± 5.1	9.3 ± 4.6	10.5 ± 5.4	0.115	-1.58
**CTQ emotional abuse >13**	80 (31.4%)	23.1%	33.3%		
**CTQ physical abuse**	7.3 ± 4.2	7.1 ± 3.7	7.5 ± 4.6	0.881	-0.15
**CTQ physical abuse >10**	52 (20.4%)	21.1%	25.0%		
**CTQ sexual abuse**	7.1 ± 5.0	5.8 ± 3.3	8.0 ± 5.9	**0.000342[0.002]**	-3.60
**CTQ sexual abuse >8**	42 (16.5%)	6.5%	25.0%		
**CTQ emotional neglect**	12.4 ± 5.8	11.9 ± 6.1	12.8 ± 5.5	0.138	-1.48
**CTQ emotional neglect >15**	88 (34.5%)	33.3%	37.7%		
**CTQ physical neglect**	7.6 ± 3.5	7.5 ± 3.4	7.7 ± 3.5	0.654	-0.45
**CTQ physical neglect >10**	50 (19.6.%)	18.5%	21.7%		

Data of scores represent means ± SD, Data of abuse according to cut-off scores are presented in total and percentage, p-value in squared bracket = FDR corrected p-value, CTQ (childhood trauma questionnaire)

According to the predefined cut-off scores of the CTQ subscales (listed in Table 2), 31.4% experienced emotional abuse, 20.4% had to face physical abuse and 16.5% were sexually abused during childhood. The mean CTQ sexual abuse score of females differs significantly from males, with higher scores in females (p<0.001, see Table 2). A total of 34.5% of patients were emotionally neglected and 19.6% physically neglected.

### 3.2 Sociodemographic risk factors

To assess sociodemographic risk factors in our sample, we performed a two-sided Fisher’s exact test for the three investigated suicidal phenotypes suicide attempt, self-harm and non-suicidal self-injury (NSSI). As depicted in Table 3, unemployment appeared as risk factor for suicide attempt (p = 0.022), not resisting FDR correction (p_corr_ = 0.06). Being single/not married did not reach significance (p = 0.051). As for self-harm, being single/not married scored significantly (p = 0.018), but also not resisting FDR correction (p_corr_ = 0.054). Both being single/not married (p = 0.035, p_corr_ = 0.052) and female (p = 0.022, p_corr_ = 0.06) showed significant p-values analysing NSSI (non-suicidal self-injury) at first sight, but lost significance after FDR correction.

**Table 3:** Sociodemographic risk factors.

	suicide attempt	self-harm	NSSI
Single/not married	χ2 = 3.72, p = 0.051	χ2 = 6.09, p = 0.018	χ2 = 5.10, p = 0.035
female	χ2 = 2.16, p = 0.158	χ2 = 2.27, p = 0.114	χ2 = 5.62, p = 0.022
unemployed	χ2 = 5.34, p = 0.022	χ2 = 0.01, p = 1.0	χ2 = 1.66, p = 0.24

p-values in squared brackets = FDR corrected p-value

To validate these results, we tested for confounders in a binary logistic regression model, with the above mentioned variables as covariates and the suicidal phenotypes as dependent variables. For suicide attempt the factors “being single” and “unemployed” contributed significantly to the model (being single: p = 0.048, OR = 1.7, 95% CI [1.01; 3.13]), being unemployed: p = 0.028, OR = 1.9, 95% CI [1.07; 3.55]). The factor female did not contribute significantly (p = 0.12, OR = 0.63, 95% CI [0.35; 1.13]).

For self-harm, “being single” was a significant risk factor (p = 0.009, OR = 2.1, 95% CI [1.18; 3.41]). The covariates female (p = 0.085, OR = 0.6, 95% CI [0.36; 1.06]), and unemployment (p = 0.95, OR = 0.98, 95% CI [0.57;1.68]) were not significant.

For NSSI, being “female” (p = 0.017, OR = 0.4, 95% CI [0.21; 0.85]) could be determined as a protective factor and “being single” (p = 0.014, OR2.3, 95% CI [1.18; 4.54]) could be determined as a risk factor. Unemployment did not have a significant impact in this model (p = 0.13, OR = 0.6, 95% CI [0.31; 1.17]).

### 3.3 Sex differences in suicide attempters in dependence of childhood trauma

In suicide attempters (n = 70, 25 male and 45 female), 82.9% were suffering from MDD and 17.1% from BD. Of male suicide attempters, 80% were suffering from MDD and 20% from BD, of female suicide attempters, 84.4% were suffering from MDD and 15.6% from BD.

In non-suicide attempters (n = 185, 85 male and 100 female), 82.7% were suffering from MDD and 17.3% from BD. Of male non-suicide attempters, 82.2% were male suffering from MDD and 17.8% from BD, of female non-suicide attempters, 82% were suffering from MDD and 17% from BD.

Comparing the mean CTQ scores (total and subscores) of patients with and without a history of suicide attempt, significant p-values could be observed for all CTQ scores in the total sample (see Table 4) and in females (see Table 5). In the total sample, significant p-values were found in all subscales (p = ≤0.018), especially in the subscales sexual abuse and physical abuse (p≤0.001). In gender-specific sub-analyses of CTQ scores, analysis of the subscale sexual abuse showed a p-value of 0.046 in males, but not resisting FDR correction (p_corr_ = 0.276). In males, no significant differences between suicide attempters and non-suicide attempters could be observed in all other subscales as well, whereas all subscales were significant in females (p≤0.021, see Table 5).

**Table 4:** CTQ scores of patients who had or had not attempted suicide, total sample.

	suicide attempters (n = 70)	non-suicide attempters (n = 185)	p-value	U
**CTQ total score**	61.1±22.9	49.2±18.1	**0.000028 [0.000084]**	-4.069
**CTQ emotional abuse**	11.3±5.4	9.5±4.9	**0.008 [0.0096]**	-2.64
**CTQ physical abuse**	8.7±5.1	6.8±3.7	**0.001 [0.002]**	-0.53
**CTQ sexual abuse**	9.5±7.2	6.2±3.6	**0.000003 [0.000018]**	-3.887
**CTQ emotional neglect**	14.2±5.9	11.8±5.6	**0.004 [0.006]**	-2.87
**CTQ physical neglect**	8.5±4.0	7.3±3.2	**0.018 [0.018]**	-2.35

**Table 5:** CTQ scores of patients who had or had not attempted suicide, males and females separately.

	suicide attempters (n = 70)		non-suicide attempters (n = 185)					
	male (n = 25)	female (n = 45)	male (n = 85)	female (n = 100)	p—male vs. male	U	p—female vs. female	U
**CTQ total score**	53.9±23.1	65.4±21.9	48.2±16.7	50.2±19.3	0.24 [0.48]	-1.176	**0.000084 [0.0005404]**	-4.404
**CTQ emotional abuse**	9.8±4.8	12.2±5.6	9.2±4.6	9.7±5.2	0.577 [0.69]	-0.558	**0.006 [0.0072]**	-2.737
**CTQ physical abuse**	8.0±4.6	9.1±5.3	6.8±3.3	6.8±4.0	0.139 [0.41]	-1.479	**0.001 [0.002]**	-3.327
**CTQ sexual abuse**	7.0±5.4	10.9±7.8	5.5±2.4	6.7±4.3	**0.046** [0.276]	-1.992	**0.000091 [0.000273]**	-3.365
**CTQ emotional neglect**	12.6±7.2	15.1±4.9	11.7±5.8	11.8±5.5	0.705 [0.705]	-0.378	**0.001 [0.002]**	-2.03
**CTQ physical neglect**	8.1±4.0	8.7±4.1	7.3±3.3	7.3±3.2	0.428 [0.64]	-0.792	**0.021 [0.021]**	-1.18

Data represent means ± SD, MDD (Major Depressive Disorder), BD (bipolar disorder), CTQ (childhood trauma questionnaire), p-values in squared bracket = FDR corrected p-values.

### 3.4 Sex differences in self-harm behaviour as a function of childhood trauma

In the examined sample 91 patients (33 male and 58 female) were presenting with self-harm behavior. In this specific subgroup 88.9% were suffering from MDD and 11.1% from BD. Of male self-harmers, 84.9% were suffering from MDD and 15.1% from BD, of female self-harmers, 89.6% were suffering from MDD and 10.4% from BD.

In non-self-harmers (n = 164, 76 male and 88 female), 79.4% were suffering from MDD and 20.6% from BD. Of male non-self-harmers, 79.6% were suffering from MDD and 20.4% from BD, of female non-self-harmers, 79.3% were suffering from MDD and 20.6% from BD.

Comparing the groups of patients with and without self-harm behaviour, significant associations (p<0.001) were found with the CTQ total score, as well as with all CTQ subscores except the sexual abuse subscore (see Table 6). The strongest associations were found with the CTQ emotional abuse and emotional neglect subscores (p<0.001). When performing gender-specific analyses, these significant associations were found in females only (see Table 7), with the strongest associations found with the CTQ emotional abuse.

**Table 6:** CTQ scores of patients with and without self-harm behavior, total sample.

	self-harm behaviour LPC (n = 91)	no self-harm (n = 164)	p-value	U
**CTQ total score**	59.5±22.5	48.6±17.7	**0.000044 [0.000264]**	-4.087
**CTQ emotional abuse**	11.6±5.5	9.1±4.7	**0.000052 [0.000153]**	-4.045
**CTQ physical abuse**	8.4±5.2	6.7±3.4	**0.011 [0.0132]**	-2.54
**CTQ sexual abuse**	8.2±6.3	6.5±4.1	0.096 [0.096]	-1.663
**CTQ emotional neglect**	14.1±5.9	11.5±5.6	**0.00085 [0.0017]**	-3.351
**CTQ physical neglect**	8.3±3.8	7.2±3.2	**0.009 [0.0135]**	-2.601

**Table 7:** CTQ scores of patients with and without self-harm behavior, males and females separately.

	self-harm behaviour LPC (n = 91)		no self-harm (n = 164)					
	male (n = 33)	female (n = 58)	male (n = 76)	female (n = 88)	p—male vs. male	U	p—female vs. female	U
**CTQ total score**	53.5±20.6	62.9±23.0	47.9±17.3	49.4±18.2	0.06	-1.879	**0.000206 [0.000618]**	-3.770
**CTQ emotional abuse**	10.3±5.1	12.4±5.6	8.9±4.4	9.2±4.9	0.107	-1.61	**0.0002 [0.0012]**	-3.718
**CTQ physical abuse**	8.0±4.9	8.7±5.5	6.7±3.0	6.7±3.7	0.177	-1.35	**0.03** [0.036]	-2.167
**CTQ sexual abuse**	6.5±4.8	9.1±6.9	5.6±2.5	7.3±5.0	0.509	-0.66	0.247 [0.247]	-1.157
**CTQ emotional neglect**	13.0±6.3	14.7±5.7	11.5±6.0	11.5±5.0	0.24	-1.176	**0.001 [0.002]**	-3.243
**CTQ physical neglect**	7.7±3.6	8.6±4.0	7.4±3.4	7.1±3.1	0.454	-1.749	**0.008 [0.012]**	-2.653

Data represent means ± SD, MDD (Major Depressive Disorder), BD (bipolar disorder), CTQ (childhood trauma questionnaire), p-values in squared bracket = FDR corrected p-values.

### 3.5 Sex differences in non-suicidal self-injury as a function of childhood trauma and depression scores

Analysis of the data in concerns of non-suicidal self-injury, 48 patients (13 male and 35 female) showed a history of non-suicidal self-injury. In this group 87.5% were suffering from MDD and 12.5% from BD. Within males with NSSI, 83.3% were suffering from MDD and 16.7% from BD, within females with NSSI, 88.6% were suffering from MDD and 11.4% from BD.

In the group of patients presenting no history of NSSI (n = 207, 97 male and 110 female), 81.6% were suffering from MDD and 18.4% from BD. A total of 81.4% of males in this group were diagnosed with MDD and 18.6.4% with BD, 81.8% of females with no history of NSSI were suffering from MDD and 18.2% from BD.

Patients with NSSI show significantly higher CTQ total scores (p = 0.001), as well as higher scores on the CTQ emotional abuse subscore (p<0.001, see Table 8). Gender specific analyses again indicate that the significant associations were due to females, but not males (see Table 9).

**Table 8:** CTQ scores of patients with and without Non-Suicidal Self-Injury (NSSI), total sample.

	NSSI (n = 48)	no NSSI (n = 164)	p-value	U
**CTQ total score**	61.2±23.1	50.3±18.9	**0.000634 [0.019]**	-3,417
**CTQ emotional abuse**	13.0±5.4	9.2±4.8	**0.000001** [0.000006]	-4,858
**CTQ physical abuse**	8.1±5.4	7.2±3.9	0.237 [0.237]	-1,182
**CTQ sexual abuse**	8.8±5.1	6.7±4.5	0.054 [0.108]	-1,924
**CTQ emotional neglect**	13.9±6.3	12.1±5.6	0.066 [0.099]	-1,842
**CTQ physical neglect**	8.2±3.6	7.5±3.5	0.123 [0.147]	-1,544

**Table 9:** CTQ scores of patients with and without Non-Suicidal Self-Injury (NSSI), males and females separately.

	NSSI (n = 48)		no NSSI (n = 164)					
	male (n = 13)	female (n = 35)	male (n = 97)	female (n = 110)	p—male vs. male	U	p—female vs. female	U
**CTQ total score**	54.8±26.1	63.7±21.7	48.8±17.1	51.8±20.3	0.335	-0.964	**0.0013** [0.0039]	-3.125
**CTQ emotional abuse**	11.8±5.5	13.5±5.4	9.0±4.5	9.5±5.1	**0.033** [0.099]	-2.133	**0.000035** [0.00021]	-4.137
**CTQ physical abuse**	8.0±5.5	8.1±5.1	7.9±3.4	7.3±4.4	0.465	-0.731	0.367 [0.367]	-0.901
**CTQ sexual abuse**	6.5±5.5	9.7±6.7	5.8±2.9	7.5±5.5	0.822	-0.225	0.117 [0.175]	-1.568
**CTQ emotional neglect**	12.4±7.4	14.4±5.8	11.8±5.9	12.2±5.3	0.962	-0.047	0.051 [0.102]	-1.955
**CTQ physical neglect**	7.9±4.3	8.3±3.3	7.4±3.3	7.5±3.6	0.746	-0.324	0.125 [0.15]	-1.536

Data represent means ± SD, MDD (Major Depressive Disorder), BD (bipolar disorder), CTQ (childhood trauma questionnaire), p-values in squared bracket = FDR corrected p-values.

## Discussion

In this study, we examined possible associations between childhood abuse and precisely defined suicidal phenotypes including suicide attempt, self-harm, Non-Suicidal Self-Injury (NSSI) and suicidal thoughts in patients suffering from MDD or BD.

The hypothesis we tested in the context of the current study was that patients suffering from MDD or BD who presented with suicidal behaviour (attempt, self-harm) and/or NSSI were exposed to more traumatic experiences in childhood.

The major finding of this study was that patients presenting with different phenotypes of suicidal behaviour show different patterns of adverse events in childhood. In detail, suicide attempters (lifetime) score significantly higher in all five subscales of the CTQ (emotional abuse, physical abuse, sexual abuse, emotional neglect, physical neglect), with highest significance levels found for the CTQ subscore sexual abuse. This pattern changes examining patients with history of self-harm. In this group of patients, as compared to the control group, the CTQ subscore sexual abuse looses significance whereas emotional abuse and neglect scores were significantly higher. Obviously, at least in our sample, childhood sexual abuse is a risk factor for suicide attempts, however not for self-harm in adulthood. The latter group, affective disorder patients with self-harm behaviour, are more likely to have had experienced emotional abuse and/or neglect during childhood. A similar pattern was found in patients with NSSI, emotional abuse scores were significantly higher than in patients without NSSI, whereas all other CTQ subscores did not differ between groups.

As mentioned before, patients with a history of suicide attempt had significantly higher CTQ scores (total CTQ score, as well as all CTQ subscales). A more precise analysis of the data reveals that the majority of these significant differences in CTQ scores derived from the female participants in our study. In males, we only found significant associations between the experience of childhood sexual abuse and a history of both suicide attempts and NSSI, however not resisting FDR correction. Generally, sexual abuse seems to have the highest impact on suicide attempts. This is partly consistent with previous research: Sarchiapone et. al. showed differences between suicide attempters and non-attempters for the total CTQ score, emotional abuse and neglect, however differences between sexes were not significant, possibly due to the smaller sample size in their study (108 patients).[14] Childhood sexual abuse (CSA) has been more frequent in females of our study cohort, which is also in line with international findings. [31] It has been shown, that CSA is associated with psychiatric disorders in adulthood and increases risk for suicide. [32, 33] CSA might be largely underestimated in males, since public attention (teachers, pediatricians) mainly focuses on girls. [32] Additionally, males often do not report sexual abuse in interviews fearing stigmatisation, vulnerability and loss of masculinity. [34]

Emotional abuse seems to play an important role with regard to self-harm behaviour (combining suicidal and non-suicidal intention). Especially female patients with a history of self-harm have significantly higher scores in the subscales of emotional abuse and emotional neglect. Similar results can be found for NSSI, where emotional abuse scores are higher in the affected group. For both, self-harm and NSSI, females show highly significant associations with the CTQ subscore of emotional abuse as well as with total CTQ score, although no differences were found in males.

This indicates that females are more sensitive to childhood abuse, with a higher risk to result in suicidal behaviour as compared to males. The gender differences in childhood maltreatment found in our study are generally consistent with findings that suggest that females might be more likely to experience abuse and neglect in childhood. [35] Our finding that childhood trauma, especially emotional abuse, is a risk factor for NSSI is partly consistent with previously published data by Swanell et al [36], who found that childhood trauma is strongly associated with the development of subsequent non-suicidal injury. In their study, childhood physical abuse correlated with NSSI in adult life, which is contrary to our results, showing low impact of physical abuse on NSSI. This discrepancy might be explained by differences in the homogeneity of the study cohorts. Our study was performed within a group of patients with an affective disorder diagnosis (MDD or BD), whereas Swanell et al randomly recruited participants in the Australian population. Weismoor et. al postulated that only those victims of physical assault show higher rates of NSSI who report more a negative self-view and greater cognitive errors (like tendency to catastrophise or overgeneralise). [37]

An explanation for gender differences found in all phenotypes might be the fact that recruitment for our study was performed in a hospital setting mainly. As shown by Mandelli et al. [10], male prisoners with a history of childhood abuse show higher risk for suicidal behaviour as well as for psychiatric disorders and aggressive traits. Especially sexual abuse increases the risk for multiple suicide attempts and other self-harm behaviour. Our findings, in conjunction with the findings from Mandelli et al., indicate that females who suffered from childhood abuse tend to react in an autoaggressive way, whereas males express their aggression towards others, often resulting in criminal behaviour and imprisonment. It has been shown that committing a violent crime is associated with male gender, younger age, greater childhood sexual abuse (CSA) and greater childhood emotional abuse. [38] This fits the concept of male depression, where externalising depression symptoms like substance use, risk-taking behaviour and aggression are in the center of attention.[39, 40] In addition, gender differences with regard to symptoms related to lowered impulse control (anger attacks) in depressed patients have been described. [41] These gender differences might also result in different approaches for treatment of depression in males, since testosterone increases serotonin transporter binding and has effects in the limbic system. [42, 43] In this context one has also to keep in mind that suicide attempts are more frequent in women, however completed suicide is more frequent in men. [7] Additionally, male suicide attempters are often misdiagnosed in the health care system and may face rejection due to the alexithymic inability to ask for help together with atypical depressive symptoms. [44] Females rather seek professional help than males when suffering from depressive symptoms. Obviously help seeking behaviour objects male stereotypes, thus leading to loss of status and being associated with loss of autonomy and self-control. [45]

The possible selection of males with childhood abuse into corrective facilities, and the fact that females with childhood abuse are rather found in clinical settings, could be an explanation for the finding, that the male sample in our study reported a rather low prevalence of childhood abuse. Another limitation includes the fact that CTQ is a self-reported questionnaire with a possible underreporting of childhood abuse experiences by males and to a lower degree also among females.

A further limitation of the current study is that we concentrated on distal triggers (adverse childhood events) of suicidal phenotypes, lacking proximal factors like traumatic events in adulthood and recent lifetime events.

The possible confounder of comorbidities leading to suicidal behavior was reduced by excluding patients with a primary organic disease.

Strength of our study is that we studied these different overlapping phenotypes of suicidal behaviour in a homogenous sample of hospitalised patients with diagnosed MDD or BD indicating the clinical importance of this work. Bias was minimised since only a small group of raters performed patient interviews.

In conclusion, we found that all phenotypes of suicidal behaviour (suicide attempt, self-harm, NSSI and suicidal thoughts) investigated it the current study showed associations with higher childhood trauma scores. Particularly, sexual abuse in childhood seems to be an important risk factor for suicide attempts, whereas patients who experienced emotional abuse during childhood seem to be at a higher risk for self-harm behaviour in later life. These findings are important arguments for a careful clinical examination of psychiatric patients presenting with self-harm in terms of early childhood trauma and the clinical approach to these closely linked phenomena.
